# *Niallia Circulans* mediated Bio-fabrication of ZnO nanoparticles: Synthesis, characterization, and multifunctional applications

**DOI:** 10.1371/journal.pone.0360387

**Published:** 2026-10-12

**Authors:** Kiran Prasad, Bhakti Patel, Gautam Priyadarshi, Dinesh Bhatia, Rakesh Kumar Verma, Esha Rami, Dipak Kumar Sahoo, Snehal Bagatharia, Ashish Patel

**Affiliations:** 1 Department of Life Sciences, Parul Institute of Applied Sciences, Parul University, Vadodara, Gujarat, India; 2 Department of Life Sciences, Hemchandracharya North Gujarat University, Patan, Gujarat, India; 3 Department of Biomedical Engineering, North Eastern Hill University, Shillong, Meghalaya, India; 4 Department of Biosciences, School of Liberal Arts and Sciences, Mody University of Sciences and Technology, Lakshmangarh, Sikar (Rajasthan), India; 5 Independent Researcher, Ames, Iowa, United States of America; 6 Gujarat State Biotechnology Mission (GSBTM), Udyog Bhavan, Gandhinagar, Gujarat, India; 7 Gujarat Biotechnology Research Centre (GBRC), M. S. Building, Gandhinagar, Gujarat, India; Universiti Tun Hussein Onn Malaysia, MALAYSIA

## Abstract

The current study addresses increasing global problems (agricultural sustainability, environmental contamination, and antimicrobial resistance) by means of sustainable and multifunctional biosynthesized Zinc oxide nanoparticles (ZnO NPs). The study utilized a cell-free supernatant of a novel endophytic bacterial strain (*Niallia Circulans*) to synthesize ZnO NPs. Biosynthesized NPs were evaluated by using various techniques of characterization. Surface plasmon resonance (SPR) was revealed by UV-Visible spectral peak for ZnO NPs at 355 nm. A X-ray diffraction (XRD) analysis confirmed a crystallite size of 90.64 nm, while scanning electron microscopy with energy dispersive X-ray spectroscopy (SEM-EDX) demonstrated the predominance of hexagonal-structured zinc, carbon, and oxygen elements. The functional groups in Fourier transform infrared spectroscopy (FTIR) confirm the presence of biomolecules. The evaluated multifunctional application of biosynthesized ZnO NPs exhibits an enhanced early seedling growth rate and increased seed germination (100% in 12 h) on cowpea (*Vigna unguiculata L.*) compared to the control groups. Also shows excellent antibacterial activity against test bacterial strains. Additionally, a high removal efficiency of 97.85% was achieved within 110 min for 10 ppm methylene blue dye solution at pH 9. This study highlights the endophytic bacteria-mediated successful synthesis of ZnO NPs, with potential multifunctional applications in agriculture, antimicrobial treatments, and environmental remediation.

## Introduction

Traditional agriculture practices largely depend on the use of agrochemicals. In parallel, increasing industrialization leads to increased pollutant discharge, causing the accumulation of carcinogenic and bacterial contaminants in the ecosystem [1,2]. These rapidly growing issues require immediate attention and resolution through innovative solutions that can simultaneously lead to sustainable agriculture and a clean environment. Synthetic dyes are widely used in textiles, pharmaceuticals, and healthcare [3]. The effluents contain highly toxic dyes, such as methylene blue (MB) dye, which is carcinogenic and mutagenic at very low concentrations [4]. MB dye can cause numerous health problems like skin irritation, toxicity on the liver and kidneys, and many more among humans. Such toxins accumulate in water resources and exhibit resistance to biodegradation, disrupting the underwater ecosystem and increasing the risk to organisms that depend on these resources [5]. These purposes aim to develop innovations that are both multifunctional and sustainable [6].

Nanomaterials possess diverse characteristics and impressive functional properties, making them highly suitable for multifunctional applications [7]. The controlled delivery of fertilizers and other drugs or molecules can be easily facilitated by using nanoparticles, as they have the potential to function as very efficient carriers, helping the living system. For example, they can directly facilitate the delivery of essential nutrients to the plant roots, thereby enhancing the overall growth of the plant by increasing the efficacy of nutrient uptake [8]. Nanoparticles (NPs) also possess antimicrobial, adsorption, and photocatalytic properties, which have been proven to be effective for environmental purification [9]. Photocatalytic activity is determined to be effective for pollutant removal, notably in dye degradation. NPs, such as TiO_2_, CuO, and ZnO, with high band gap energy generate reactive oxygen species (ROS) in the presence of UV or visible light. They also exhibit excellent electron mobility with multiple active sites, which enhances reactivity and leads to the maximum generation of the most active hydroxyl radicals (•OH) [10,11].

Nanomaterials can be categorized into various types, including carbon nanotubes, nanocomposites, quantum dots, and metal nanoparticles (MNPs). MNPs exhibit remarkable properties, including excellent applications with high potential in various disciplines [12]. The MNPs facilitate environmental remediation due to their high surface area, which shows potential for removing various pollutants [13]. Numerous researchers have conducted studies to evaluate the applicability of NPs, particularly in cancer therapy and also as antimicrobial agents in the medical sector [14]. Among all MNPs, Zinc oxide nanoparticles (ZnO NPs) are more beneficial, as they exhibit low toxicity in living systems and also possess the natural capacity for degradation. Due to such valuable properties of ZnO NPs, they can be referred to as an ideal material to be used in the agriculture sector [15]. Zinc is well known as an essential micronutrient that significantly enhances plant growth when applied. ZnO NPs show more efficient and rapid absorption than bulk zinc due to their nanoscale size range. The studies concluded that ZnO NPs can accelerate seed germination and plant growth when applied in optimized quantities [16]. Additionally, they also exhibit antimicrobial potential against a broad spectrum of microorganisms [17]. ZnO NPs have also been proven to show excellent photocatalytic activity, which can eliminate dye contaminants from water in the presence or absence of UV light [18]. As ZnO NPs demonstrate multifunctional benefits, they can be an ideal material for applications in various sectors, including sustainable agriculture development, environmental cleanup, and as an antimicrobial agent.

The various challenges that occur during the traditional approaches (both chemical and physical methods) of ZnO NPs synthesis can be easily addressed through biogenic synthesis. Conventional methods encounter several challenges, including high energy requirements and the use of chemicals that have been proven toxic. However, the biosynthesis is comparatively simple, eco-friendly, cost-effective, and more sustainable for the synthesis of ZnO NPs [19]. The biological system-mediated synthesis, using plants, algae, bacteria, or fungi, increases the importance of biosynthesis, as it produces sustainable nanomaterials [20–23]. The biosynthesis of NPs is facilitated by the presence of various biomolecules [24–27]. Plants include the phytochemicals (like phenols, flavonoids, etc), whereas microbes also produce various reductase enzymes, and many other proteins play a crucial role in the reduction and stabilization of NPs [28–30]. The researchers reported the successful bacteria-mediated synthesis of ZnO NPs using bacteria such as *Lactobacillus plantarum*, *Rhodococcus pyridinivorans*, and *Bacillus megaterium* [31–33]. The enhanced biocompatibility and stability of biosynthesized ZnO NPs were also observed compared to those of conventionally synthesized ZnO NPs [34–36].

The symbiotic relationship-forming group of bacteria that resides within plant tissues is known as endophytic bacteria. These endophytes can produce highly impressive biomolecules that significantly enhance plant growth, making them particularly suitable for use in agriculture. These biomolecules also possess the ability to reduce zinc ions for the successful synthesis of ZnO NPs. Such endophytic bacterial-mediated synthesis of ZnO NPs can increase the efficiency in plant growth promotion. This study represents the use of the novel endophytic bacterial strain (*Niallia circulans*) for the synthesis of ZnO NPs. The synthesized ZnO NPs were confirmed to have a nano-range size and purity by evaluating them through various analytical techniques. They were examined for their potential applications in various areas, including agriculture, the purification of contaminated water, and antimicrobial activity. The novelty of the study lies in the use of microbes for the successful synthesis of ZnO NPs and their multifunctional properties, which offer excellent applications in various sectors. This study addresses the major concerns of society, including agricultural issues, dye-contaminated water systems, and microbial contaminations, by employing an integrated biological approach for the synthesis of NPs.

## Materials and methods

### Materials

Zinc acetate dihydrate [Zn (CH_3_COO)_2_·2H_2_O], Nutrient broth (n-broth), and Muller-Hinton agar were purchased from Himedia (India). Methylene blue dye and ethanol were procured from Merck (India). Certified seeds of cowpea (*Vigna unguiculata L.*) were sourced from a local agriculture supplier in Gujarat, India.

### Isolation of endophytic bacteria and their culture preparation

The endophytic bacteria (*Niallia circulans*) were isolated from the leaf of the *Fagonia sp.* plant collected in June from the non-protected desert area of Jaisalmer, Rajasthan, India, by following the same procedure as mentioned in the previous study [37]. The plant is not listed as an endangered or protected species under the Wildlife Protection Act of India (1972) or the Biological Diversity Act (2002). Hence, no special authorization or permission was required for the collection of this plant. The bacterial isolates were incubated at 37 ºC after inoculation in n-broth for 24 h. After inoculation, the cell abundance of the culture was estimated by measuring the optical density (OD) at 600 nm using a UV-Visible spectrophotometer. As soon as the OD was measured, more than 1, the culture was centrifuged at 10,000 rpm for 10 min. The collected supernatant was further used for the biosynthesis of ZnO NPs.

### Molecular analysis for identification of bacterial isolate

The genomic material (DNA) was extracted using the phenol-chloroform method and evaluated for the desired quality by 0.8% agarose gel electrophoresis, along with a UV-Visible spectroscopic technique in the range of 260–280 nm. Then, for the amplification of the 16S rRNA gene, PCR was conducted using universal primers 27F (AGAGTTTGATCAATGGCTCAG) as the forward primer and 1492R (TACGGTACCTTGTTACGACTT) as the reverse primer. After amplification, the product was sequenced using Sanger sequencing and then submitted to the NCBI GenBank database. The sequence was compared with the NCBI nucleotide database by running BLAST. The aligned sequences were used to construct a phylogenetic tree using Tamura 3-parameter model by the neighbor-joining method in MEGA (version 12.0) software.

### Biosynthesis of Zinc oxide nanoparticles (ZnO NPs)

The collected bacterial cell-free supernatant was gradually added to a freshly prepared 25 mM aqueous solution of zinc acetate dihydrate precursor at a 1:4 ratio, under continuous stirring conditions. A negative control was carried out using the same precursor under identical conditions without bacterial cell-free supernatant. After 2 h of stirring at pH 9 and 60 ºC, the control showed no formation of nanoparticles, whereas the reaction mixture that formed NPs was allowed to settle the whitish particles of ZnO NPs. The settled particles were collected and washed by centrifuging twice with ethanol and three times with deionized water to remove impurities. The washed pellets were dried overnight at 70–80 ºC in a hot air oven to remove moisture from the sample. Dried pellets containing ZnO NPs were calcinated in a muffle furnace at 400 ºC for 2 h. As soon as NPs reached room temperature, they were stored in a clean Eppendorf for further use.

### Characterization of Biosynthesized ZnO NPs

The synthesized ZnO NPs using a novel strain of endophytic bacteria (*Niallia circulans*) were characterized employing techniques like UV-Visible Spectroscopy, FTIR, XRD, and SEM-EDS.

### UV-Visible spectroscopy

The ZnO NPs synthesized biologically were examined within the wavelength spectrum of 200–800 nm at 1 nm resolution by a UV-Visible spectrophotometer (LABMAN, LMPS-UV1900S).

### Zeta potential

The zeta potential of the biosynthesized ZnO nanoparticles (ZnO NPs) was determined using the HORIBA SZ-100 zeta potential analyzer. Zeta potential study was performed at 25.1 °C, with dispersion medium viscosity and conductivity of 0.893 mPa·s and 0.312 mS/cm, respectively. The zeta potential of produced ZnO NPs was measured to be −21.4 mV, while their electrophoretic mobility was −0.000166 cm^2^/V·s.

### FTIR analysis

FTIR analysis confirms the function of biomolecules as stabilizing and reducing agents. The FTIR reveals the existence of a functional group within the analyzed material. KBr was used to form a pellet sample for FTIR analysis in the 600−4000 cm^-1^ range using a PerkinElmer FTIR spectrophotometer.

### XRD analysis

A Phase XRD-D2 powder X-ray diffractometer (Rigaku Mini flex 600) equipped with Cu-Kα radiation was used for XRD analysis. This covers a broad range of Bragg angles (3° ≤ θ ≤ 90°) at an operating voltage of 30 kV and a current of 2 mA. The equation (Debye-Scherrer’s) is utilized to calculate the average crystalline size of the material in nm as follows:


$$\begin{array}{c} \text{D}=\frac{\text{K}\lambda }{\beta \text{cos}\theta } \end{array}$$

Where, K: 0.94

λ: wavelength of X-ray radiation, λ = 1.54059 Å,

β: full width at half maximum (FWHM)

θ: diffraction angle.

### SEM-EDS analysis

The composition, morphology, and dimensions of ZnO NPs can be evaluated using SEM-EDS (FEI APREOS). SEM was operated at a working distance of 6.8 mm under high-vacuum conditions, with an energy input of 20 kV.

### Anti-bacterial activity

The antibacterial properties of the synthesized ZnO NPs were evaluated using the disc diffusion method. The prepared bacterial cultures of the test organisms (*E. coli, Bacillus cereus, Serratia marcescens,* and *Bacillus megaterium*) were swabbed onto Muller-Hinton agar plates. The sterile discs were loaded by dipping them in the (20, 40, 60 ppm) solution of ZnO NPs. Bacterial cell-free supernatant and precursor solution were used as negative controls, while a standard antibiotic was used as a positive control for comparison. The loaded disc control discs were transferred onto the swabbed plates and incubated for 24 h. After incubation, the zone of inhibition against the test strain was measured.

### Seed germination

The application of ZnO NPs was evaluated on cowpea (*Vigna unguiculata*) seeds to observe the seed germination activity. The certified cowpea seeds were weighed and cleaned with deionized water; subsequently, 5% sodium hypochlorite was used to sterilize them. Then the seeds were soaked for 2 h in deionized water, bacterial cell-free supernatant, zinc acetate solution as a control, and in different concentrations (10, 20, 30 ppm) of ZnO NPs solution. The seeds were then placed in a sterile tissue-paper-lined petri plate and kept in dark conditions to facilitate germination. Seeds were observed every 2 h up to 24 h, and germination was recorded. To calculate germination percentage (GP) following formula was used:


$$\begin{array}{c} \text{GP}=\;\frac{\text{seeds germinated}}{\text{total seeds soaked}\times \text{100}} \end{array}$$

Germinated seeds were weighed to get the fresh weight of seeds, and then, using the following equation water content (WC) percentage was calculated:


$$\begin{array}{c} \text{WC }\%=\frac{\text{fresh weight}-\text{dry weight}}{\text{ fresh weight}}\times \text{100} \end{array}$$

### Methylene blue dye remediation

To assess the potency of ZnO NPs in remediating MB dye, an aqueous solution of 10 ppm was prepared. In 100 mL of dye solution, approximately 10 mg of ZnO NPs were added under stirring conditions in the presence of UV light. The control bacterial cell-free supernatant and zinc acetate precursor solution were separately kept under the same experimental conditions. The aliquots of dye solution were taken at 10-minute intervals to measure absorbance using a UV-Visible spectrophotometer. The formula used to calculate the percentage removal of dye was as follows:


$$\begin{array}{c} \%\text{ Dye Removal}=\frac{\left({\text{C}}_{\text{0}}-{\text{C}}_{\text{t}}\right)}{{\text{C}}_{\text{0}}\times \text{100}} \end{array}$$

Where C_0_: initial concentration, and C_t_: concentration at a particular time (t).

The kinetics of dye degradation were evaluated to determine the rate of dye degradation. The experimental data were fitted using a linear pseudo-first-order (LPFO) kinetic model. This estimates the proportionality between the rate of dye removal and the concentration of dye remaining in the solution. The following equation was use for LPFO kinetic model fitting:


$$\begin{array}{c} \text{ln}\left(\frac{q_{e}}{q_{t}}\right)=\text{ln}q_{e}-k_{\text{1}}t \end{array}$$

Where, q_e_ and q_t_ in (mg/g) indicate the quantity of MB dye degradation at equilibrium and at time t (min), K^1^ (min^-1^) is the rate constant for the LPFO model.

### Statistical analysis

All the experiments were carried out in triplicate. Statistical analysis, such as ANOVA and Student’s t-test, was applied to compare and differentiate between the control and treated groups. The results are presented as the average mean value ± standard deviation (SD), with p-value ≤ 0.05.

## Results and discussion

### Molecular analysis for identification of bacterial isolate

The bacterial isolate was identified as *Niallia circulans* (OR259009.1:1–913) through 16S rRNA gene sequencing, followed by a comparison of the sequence with the NCBI nucleotide database and the use of BLAST analysis. A phylogenetic tree was constructed using the Tamura 3-parameter model by the neighbor-joining method in MEGA (version 12.0) software, which suggests the isolate lies within the *Niallia* genus (Fig 1).

**Fig 1:**
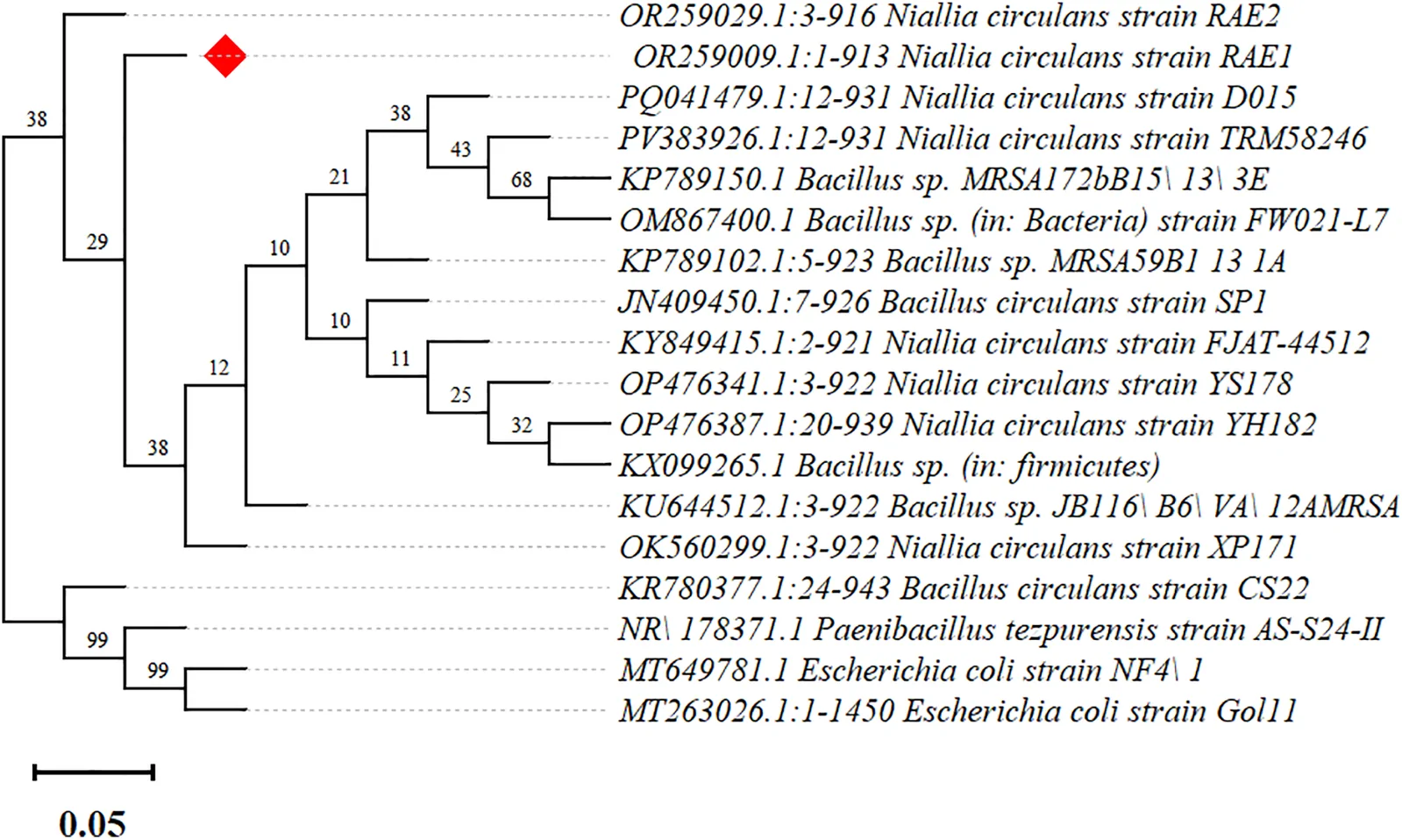
Phylogenetic tree of the bacterial isolate (*Niallia circulans*) identified by 16S rRNA gene sequencing. Neighbor-joining tree showing close relations with the *Niallia* genus with bootstrap values (1000 replicates).

The ZnO NPs were fabricated utilizing a cell-free supernatant of the isolated endophytic bacteria (*Niallia circulans*). These NPs were characterized using various techniques to confirm the formation of ZnO NPs.

### UV-Visible Spectroscopic analysis of biosynthesized ZnO NPs

The UV-Vis spectroscopy reveals the optical properties and successful formation of biosynthesized ZnO NPs. The absorbance spectrum exhibited a sharp peak at 355 nm, which corresponds to the intrinsic band-gap absorption of ZnO and gives preliminary confirmation of ZnO NPs synthesis (Fig 2). The spectra of bacterial cell-free supernatant (CFF) and zinc acetate (ZA) solution were also examined and shown in (Fig 2) as control samples for comparison. The fabrication of ZnO NPs is confirmed by the observation that neither of these control samples exhibits the absorption peak at 355 nm observed for the produced ZnO NPs. A study conducted by Aslinjensipriya et.al. also observed the same peak at 355 nm for ZnO NPs. In contrast, Kamal et al. observed an absorption band within the range of 350–380 nm, aligning well with our results [38,39]. To further evaluate the optical properties, the Tauc’s plot was constructed using the absorption coefficient versus photon energy. The Tauc curve revealed an estimated band-gap energy (Eg) of 3.22 eV (Fig 2). This value shows the blue shift of ZnO NPs compared to bulk ZnO. Hence, the band gap confirms the formation of nanosized ZnO with good optical properties.

**Fig 2:**
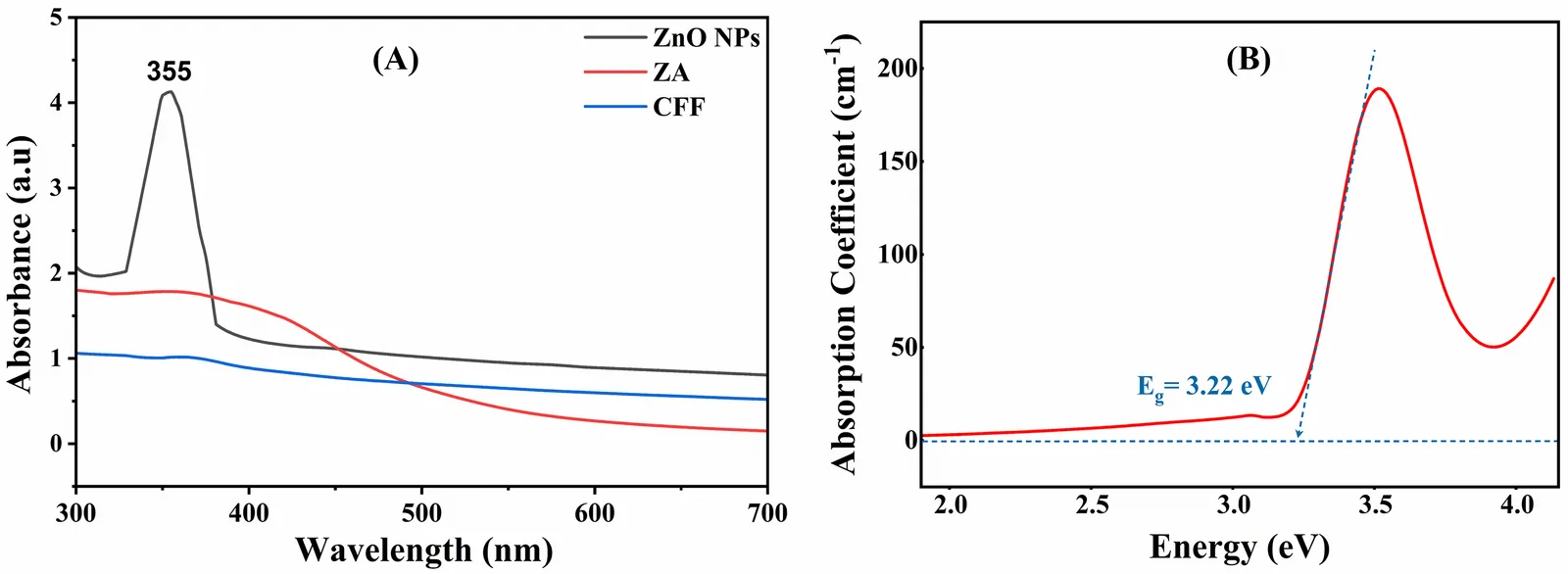
(A) UV-Visible spectrum of synthesized ZnO NPs with Zinc acetate precursor (ZA), and Bacterial cell-free supernatant (CFF), and (B) Tauc’s plot of the *Niallia circulans* mediated synthesized ZnO NPs.

### Zeta potential analysis

The resulting zeta potential of the synthesized ZnO nanoparticles was −21.4 mV, indicating that the nanoparticles had a negative charge on their surface. Electrophoretic mobility of ZnO nanoparticles was −0.000166 cm²/V·s. The measurements were taken at 25.1 °C, with a dispersion medium viscosity of 0.893 mPa·s and conductivity of 0.312 mS/cm. The negative charge on the nanoparticles’ surfaces suggests the existence of negatively charged molecules [40].

### FTIR analysis of biosynthesized ZnO NPs

The FTIR spectra offer insight into the surface chemistry of ZnO NPs and reveal the functional groups in the biomolecules that facilitate the reduction and capping of bio-reduced ZnO NPs (Fig 3). High-intensity stretching was observed at 3572 cm^-1^, indicating the presence of hydroxyl groups (OH), while stretching at 3457 cm^-1^ corresponded to amide groups (N-H) [41]. The methylene group (C-H) of proteins and carbonyl groups (C = O) of amides show stretching at 2986 cm^-1^ and 1699 cm^-1^, respectively [42,43]. The peak at 1599 cm^-1^ confirmed the presence of C = C and N-H bonds. Sulfoxide group (S = O), hydroxyl groups (OH), and methylene group (C-H) stretching was revealed by a peak at 1366 cm^-1^. Finally, peaks at 991 cm^-1^ and 774 cm^-1^ confirmed metal-ligand (ZnO) bonding [44].

**Fig 3:**
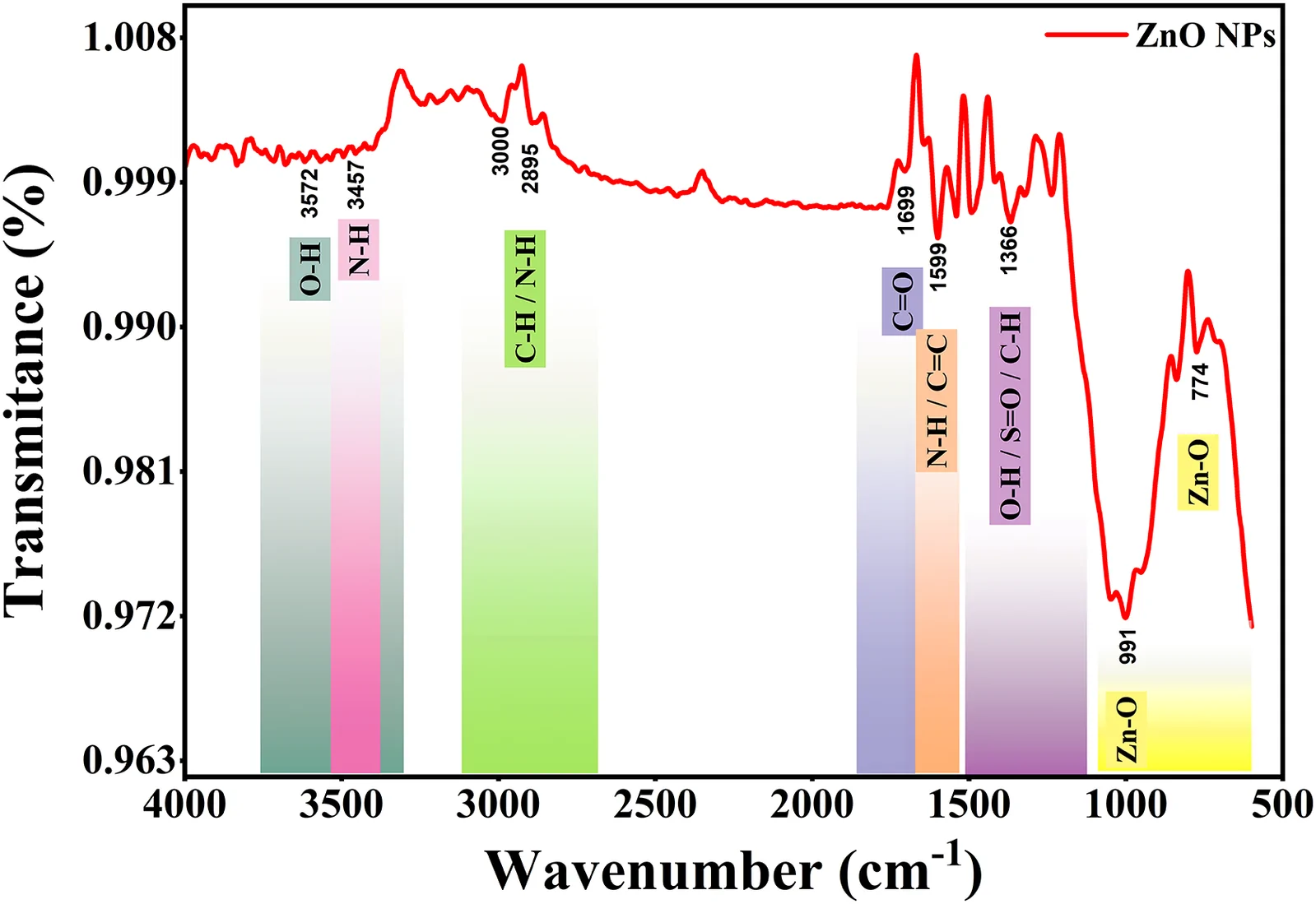
The FTIR analysis spectrum indicates the presence of various functional groups in the biosynthesized ZnO NPs sample.

### Crystalline structure analysis using X-Ray Diffraction (XRD)

The XRD of biosynthesized ZnO NPs revealed distinct crystalline peaks at specific 2θ angles (Fig 4). The observed peak positions were 31.84º, 34.16º, 36.48º, 47.94º, 56.82º, and 67.86º corresponding to the Miller planes (100), (002), (101), (102), (110), and (112), respectively [45]. The peaks other than this may have been due to the presence of minor impurities or amorphous phases. The wurtzite hexagonal structure of ZnO NPs was indicated by these peak positions and planes, consistent with the Joint Committee on Powder Diffraction Standards (JCPDS) card no. 36–1451 [46]. Using Scherrer’s equation, the calculated crystalline size was 10.54 nm for the material.

**Fig 4:**
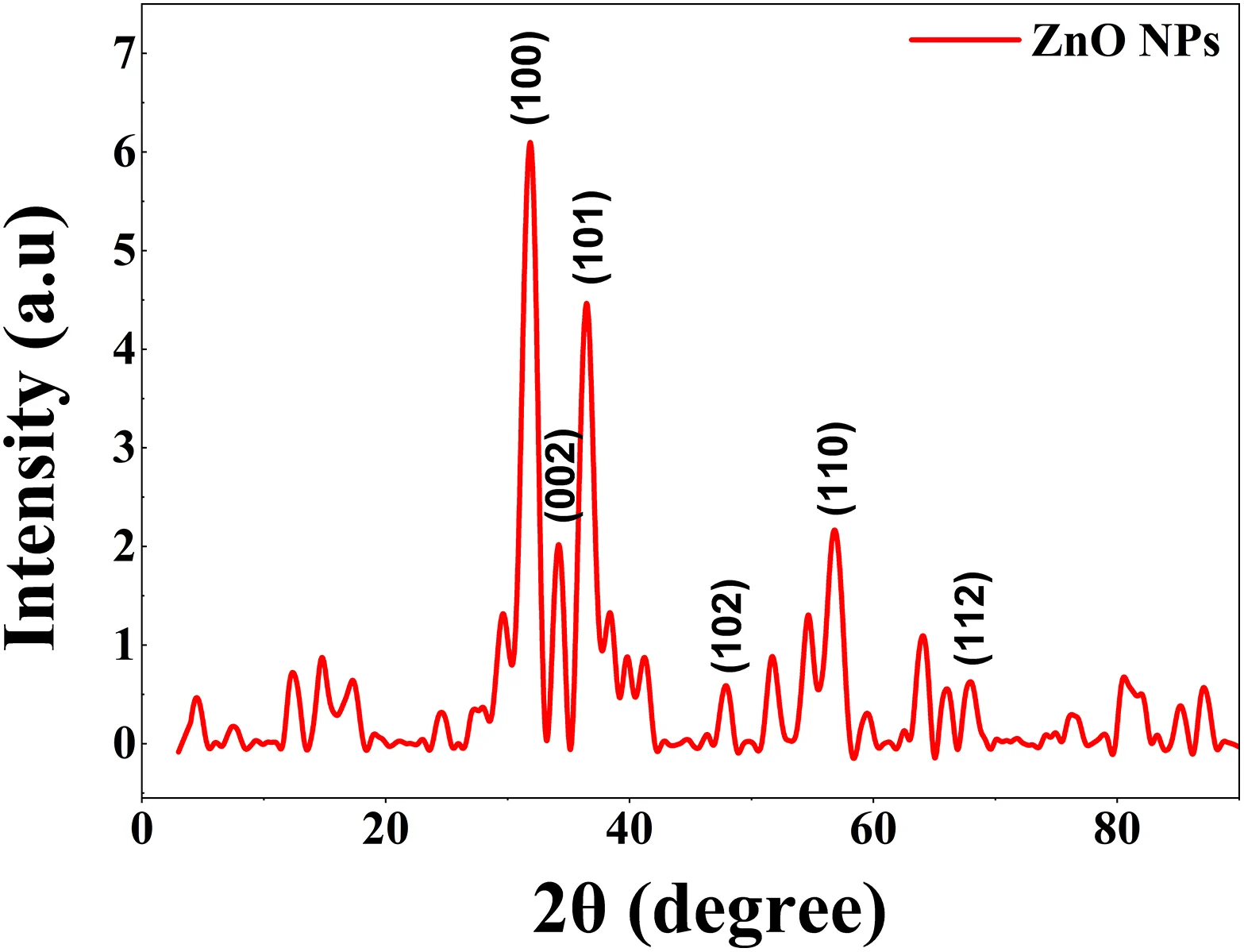
XRD diffractogram indicating the peak to determine the crystallinity of the ZnO NPs.

### Morphological Studies of ZnO NPs (SEM-EDX)

The high-resolution SEM image of biosynthesized ZnO NPs (Fig 5) reveals aggregates of hexagon-like particles with an average size of 62.52 nm. The study conducted by Saravanan et al. also observed ZnO NPs in the range of 45–95 nm [33]. EDX spectroscopy analysis of ZnO NPs samples indicates an elemental composition of 71.45% zinc, 6.24% oxygen, and 22.31% carbon. The electron mapping image confirms the presence of zinc within the material, demonstrating the exceptional purity of the ZnO NPs. A previous study conducted by Rashid et al. found closely related results 72.27% for Zn and 8.35% for O during EDX analysis of biologically synthesized ZnO NPs [47].

**Fig 5:**
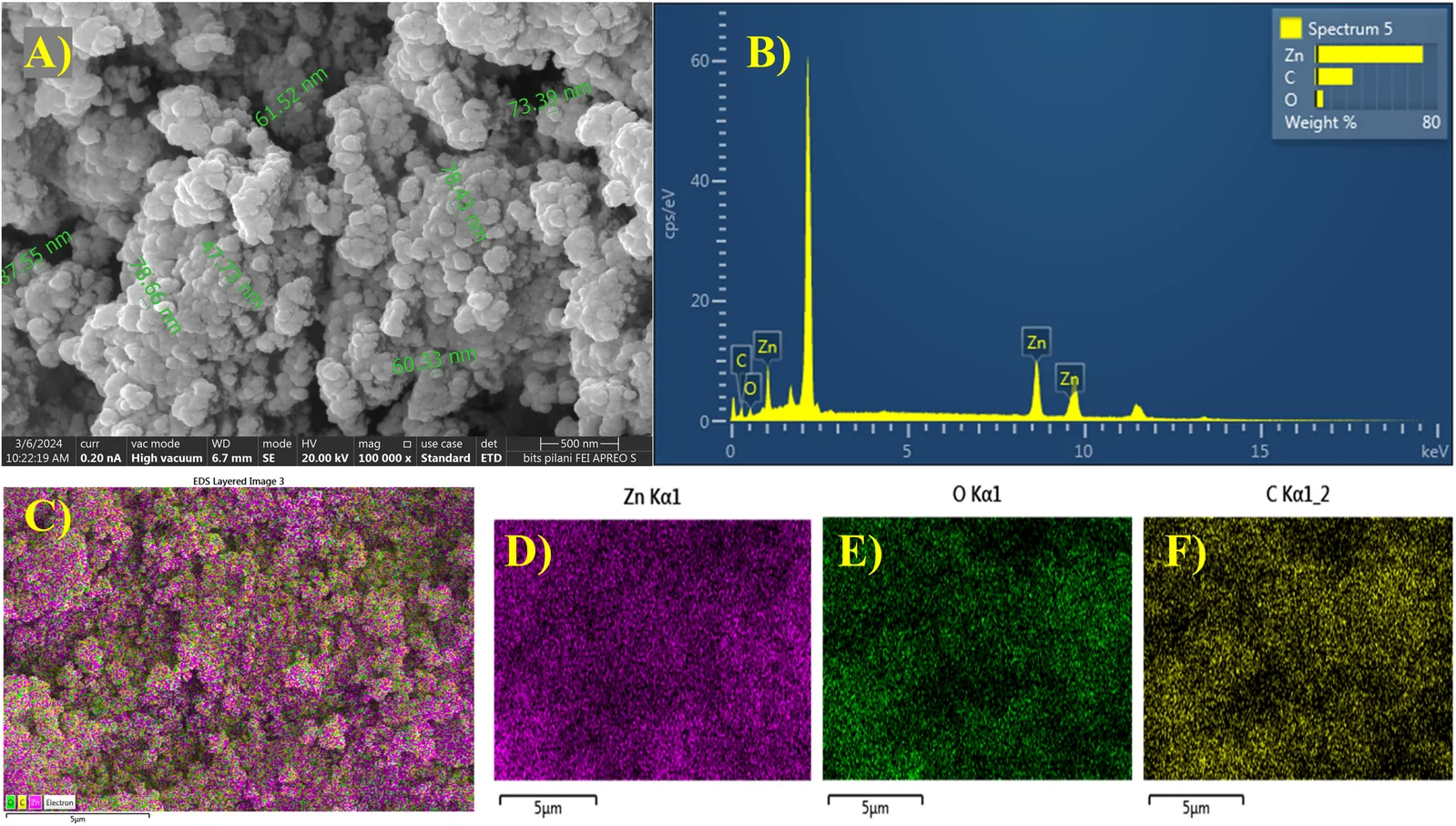
A) SEM image at 500 nm scale range, B) EDX spectrum, and C) electron mapping images of the biosynthesized ZnO NPs. Individual elemental electron mapping of D) Zinc, E) Oxygen, F) Carbon in the sample.

### Antibacterial activity

The synthesized ZnO NPs were assessed for their antibacterial properties using the disc diffusion method against both Gram-positive and Gram-negative bacterial pathogens (Fig 6). The highest concentration of ZnO NPs (60 ppm) demonstrated the most significant antibacterial effect, with *Bacillus megaterium* exhibiting the largest zone of inhibition (ZOI) at 32 mm. Lower concentrations of 40 and 20 ppm resulted in ZOIs of 17 and 14 mm, respectively, for *Bacillus megaterium*. *Bacillus cereus* showed ZOIs of 26, 20, and 12 mm at 60, 40, and 20 ppm, respectively. *Serratia marcescens* and *E. coli* displayed identical ZOIs of 15 mm at 60 ppm, 14 mm at 40 ppm, and 12 and 11 mm at 20 ppm, respectively. The control bacterial cell-free supernatant showed no ZOI (Fig 6). Zinc acetate did not show a significant ZOI during all experiments, and standard antibiotic discs showed 11, 13, 10, and 13 for *E. coli, Bacillus cereus, Serratia marcescens,* and *Bacillus megaterium,* respectively*.* These results indicate that synthesized ZnO NPs possess potent antimicrobial activity against the tested bacterial strains.

**Fig 6:**
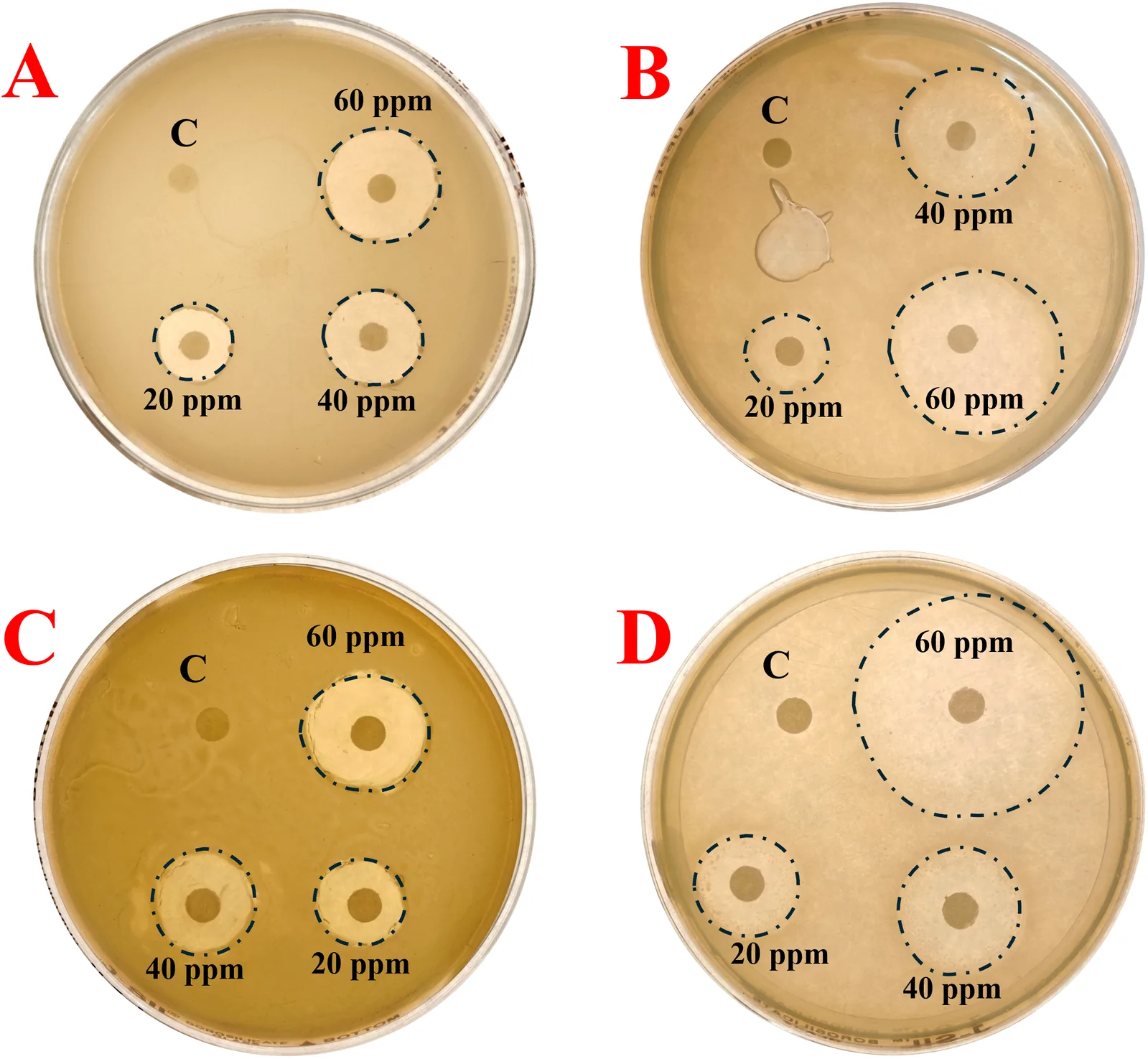
Image of antibacterial activity indicating zone of inhibition (ZOI) in:A) *E. coli,* B) *Bacillus cereus,* C) *Serratia marcescens,* and D) *Bacillus megaterium.*

Similarly, Shanmugam et al. observed approximately 9 mm ZOI against *Serratia marcescens*, while Abdullah et al. recorded 200 ppm ZnO NPs synthesized using *Pseudomonas aeruginosa,* which showed 12.33, 29.3, 19.3, and 11.7 mm ZOI against *Staphylococcus aureus*, *Bacillus subtilis*, *E. coli*, and *Pseudomonas aeruginosa,* respectively [48,49].

The antibacterial activity of ZnO NPs is likely due to multiple mechanisms, including the generation of reactive oxygen species (ROS), the release of Zn^2+^ ions, and direct contact of ZnO NPs with bacterial cell walls, which may induce damage. These may all generate a synergistic effect and contribute equally to the potent antimicrobial activity of biosynthesized ZnO NPs. The excellent cytotoxicity of such NPs has been previously reported, revealing the exact mechanism of ROS generation. These contribute to the promotion of oxidative stress and DNA damage, leading to cell apoptosis, as they share the exact mechanism. Further cytotoxicity studies of these ZnO NPs may yield promising results in the future [50,51].

### Seed germination activity in Cowpea

The Cowpea seeds were germinated in control (zinc acetate and bacterial supernatant) and treated conditions with various concentrations (10, 20, 30 ppm) of ZnO NPs. The application of ZnO NPs enhanced both the germination percentage and rate of seed germination compared to the control groups (Fig 7). The treated seeds showed 100% germination within 12 h, while the control group reached 98% germination after 16 h. After 5 days after sowing (DAS), the average root length in the 10 ppm treated group measured 3.21 ± 0.32 cm, with an average shoot length of 8.24 ± 0.36 cm. The treatments had significant effects on root length (one-way ANOVA, F(5,12) = 24.85, p < 0.001) and shoot length (F(5,12) = 94.43, p < 0.001). The post-hoc test, Tukey’s HSD, indicated that the 10, 20, and 30 ppm treatments were statistically different from the control, bacterial CFS, and zinc acetate groups. However, differences between the ZnO NP treatments were nonsignificant. However, root and shoot lengths decreased at higher ZnO NPs concentrations (20 and 30 ppm) compared to the 10 ppm treatment. However, they remained greater than those observed in the control group (Fig 8). The dose-dependent pattern of the response suggests that ZnO NPs may have a dose-specific or dual mode of action, in which lower doses confer an advantage due to increased zinc availability, while higher doses cause oxidative stress or interfere with nutrient or water uptake, inhibiting further root or shoot growth. The impact is that the doses of ZnO NPs employed were advantageous to seedling growth, however, the effect became less evident as doses increased. The study conducted by Sarkhosh et al. recorded that the application of ZnO NPs increased the 30% germination percentage compared to the control in both *Brassica napus L.* and *Camelina sativa* [16]. Similarly, Rai-Kalal et al. observed increased seed germination in wheat through biopriming with ZnO NPs [52].

**Fig 7:**
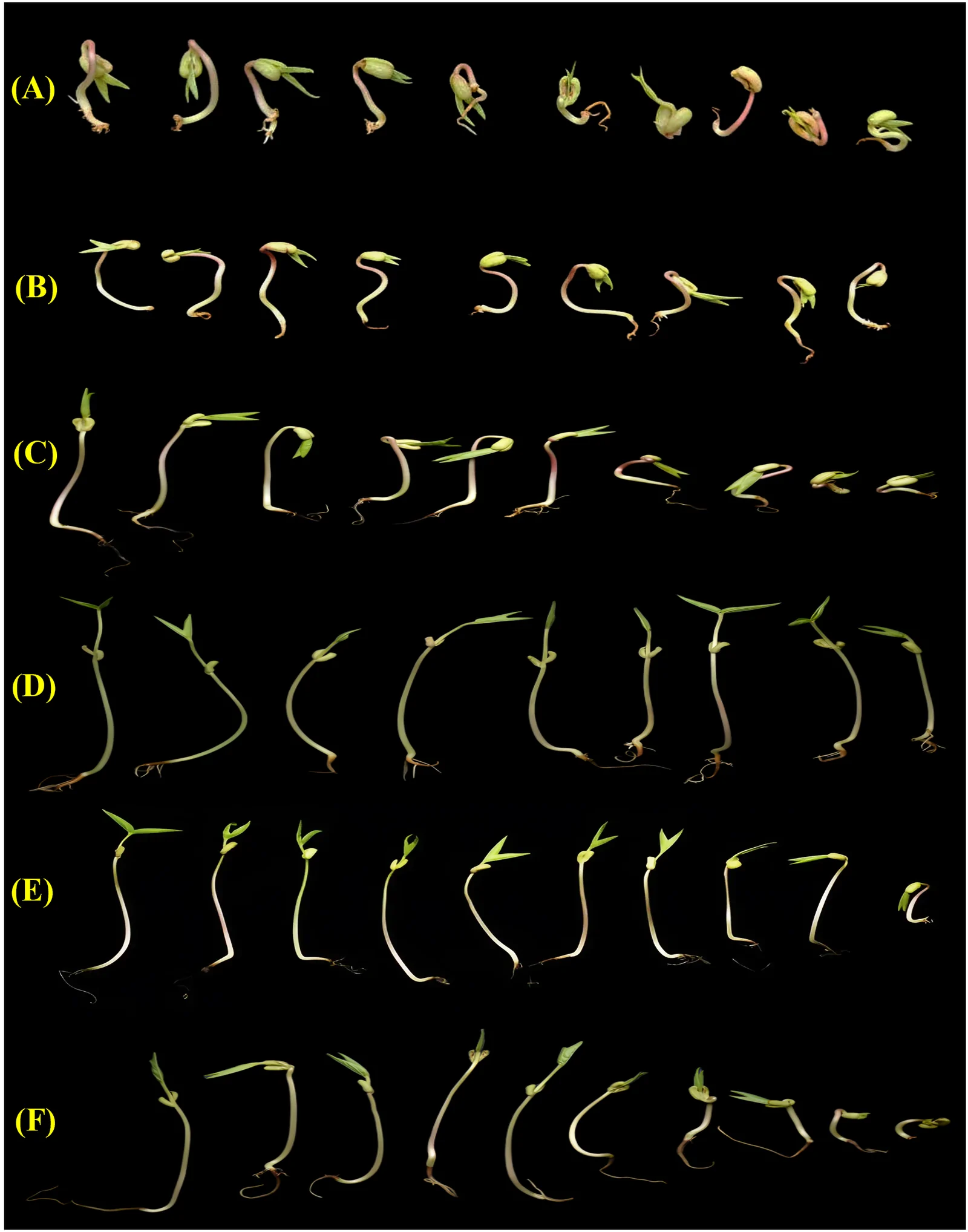
Image of (A) zinc acetate, (B) bacterial supernatant, (C) Control, (D) 10 ppm ZnO, (E) 20 ppm ZnO, (F) 30 ppm ZnO treated seedling 5 days after sowing (DAS).

**Fig 8:**
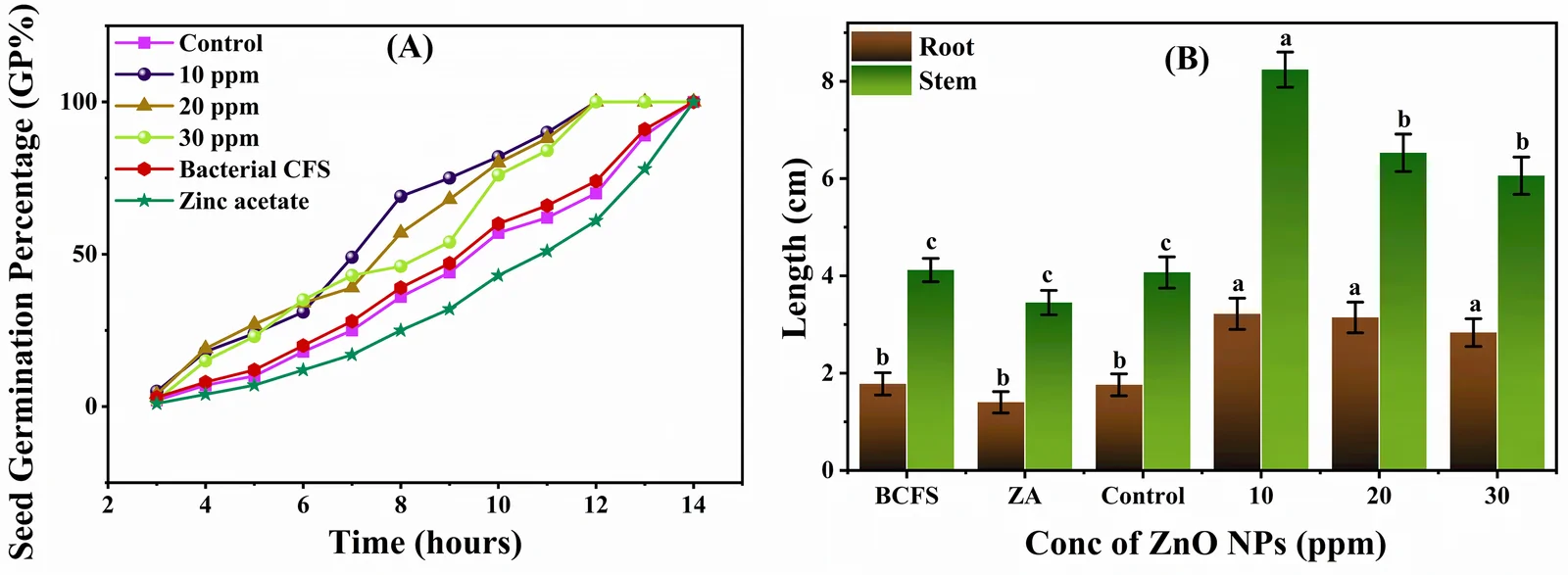
Effect of ZnO NPs on (A) seed germination percentage (GP %) within 14 hours in cowpea (*Vigna unguiculata*) and (B) their root and shoot lengths (cm) in zinc acetate (ZA), bacterial supernatant (BCFS), water control, as well as in ZnO NPs treated groups. Values are presented as mean ± SD (n = 3).

### Methylene blue dye remediation

The ZnO NPs revealed a promising result in the MB dye remediation under UV light exposure, while the bacterial cell-free filtrate and zinc acetate precursor controls showed no appreciable dye removal under the same experimental conditions, confirming that the observed remediation activity was primarily associated with the synthesized ZnO NPs. The contaminants were effectively removed in the presence of UV light, as the ZnO has a band gap energy of 3.22 eV, which increases the redox potential of NPs. The generation of hydroxide and superoxide radicals promotes the photocatalysis [53]. Absorbance measurements were recorded at 10-minute intervals, with the MB dye peak measured at a wavelength of 663 nm. A decrease in dye concentration in the sample corresponded to a reduction in absorbance. The biosynthesized ZnO NPs exhibited excellent photocatalytic activity, achieving 50% remediation within 20 min, reaching 97.85% within 110 min. The maximum remediation efficiency was achieved at a 10 ppm MB concentration and pH 9, using 10 mg of ZnO NPs (Fig 9). The complete removal of MB dye at 50 min was obtained by Venkatesan et al. using ZnO NPs synthesized from *Euphorbia milii* leaf extract [5].

**Fig 9:**
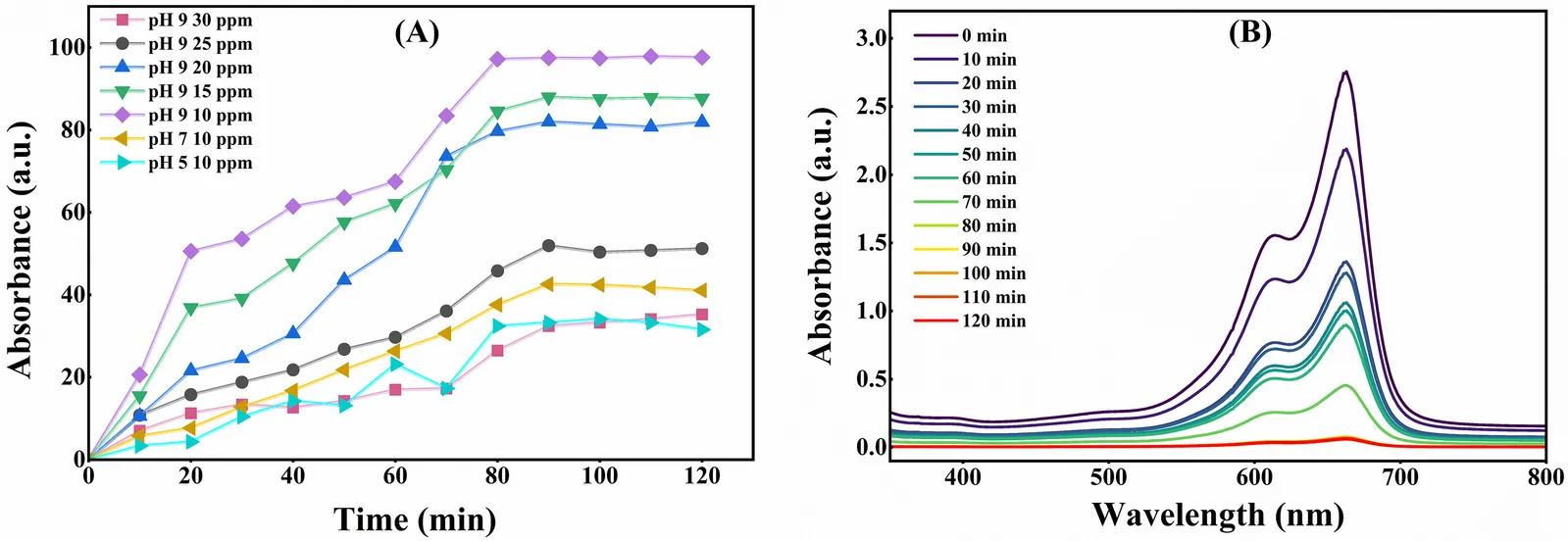
The graph of (A) MB dye removal percentage by ZnO NPs at various dye concentrations and pH (B) and 10 ppm MB dye removal spectrum at pH 9 at regular time intervals.

The linear pseudo-first-order (LPFO) kinetic model was employed to assess the rate of photocatalytic degradation of MB dye (Fig 10). The linear plot of ln(q_e_/q_t_) versus time (min) shows a straight-line graph, indicating that the degradation of MB dye follows the LPFO kinetic model. The R^2^ value obtained was 0.91, which suggests that the LPFO model fits well and accurately describes the kinetic behavior of the system.

**Fig 10:**
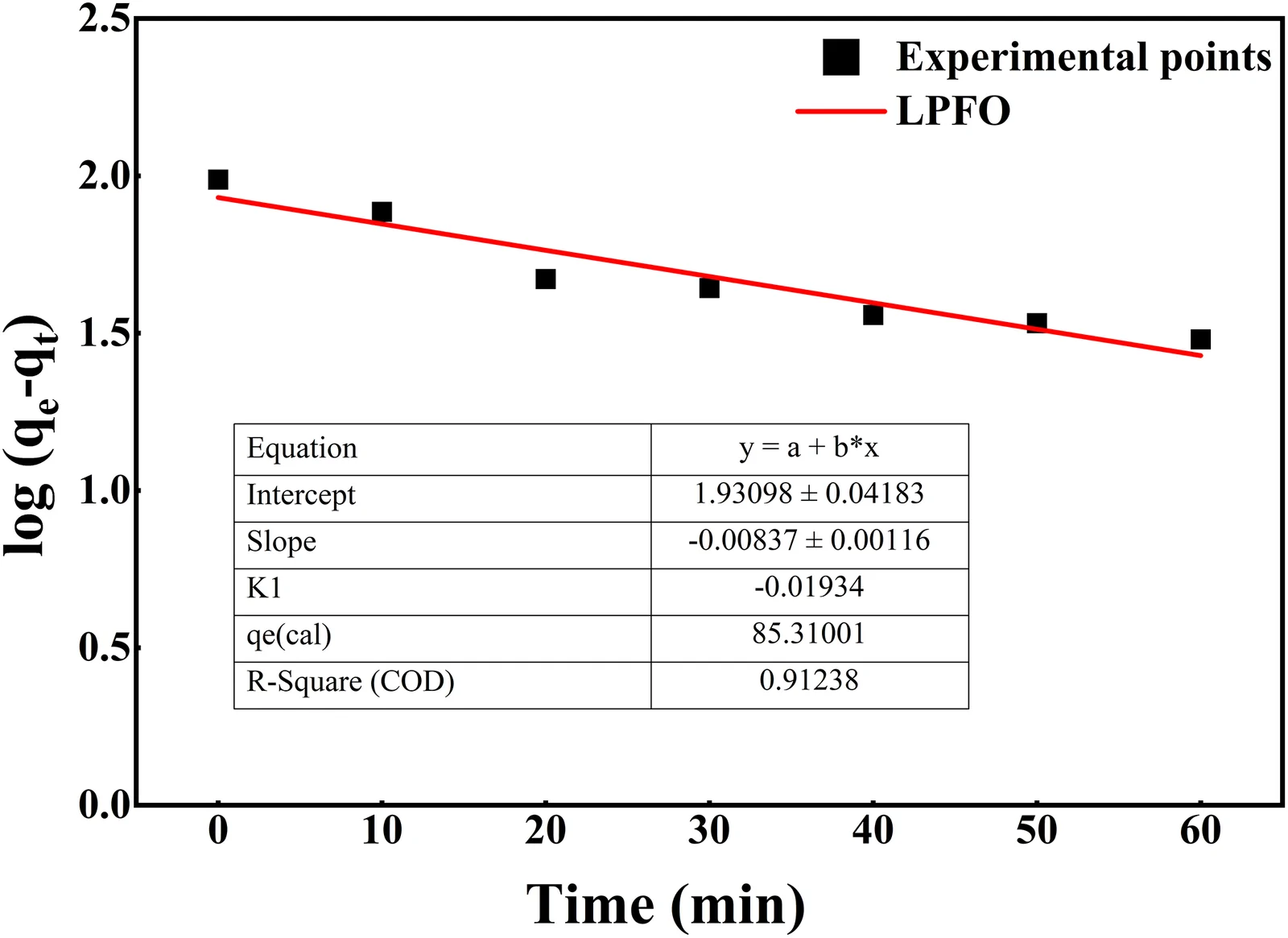
The graph of pseudo-first-order kinetic linear fitting for photocatalytic degradation of MB dye using biosynthesized ZnO NPs.

## Conclusion

In this study, ZnO NPs were successfully fabricated using an endophytic strain of bacteria (*Niallia circulans*), an easy, rapid, environmentally friendly, and cost-effective method. These biosynthesized ZnO NPs were characterized and confirmed the nanoscale formation. The multifunctional properties of ZnO NPs were evaluated, which exhibit promising results. The enhanced germination percentage in cowpea (*Vigna unguiculata L.*) was accompanied by an increase in root and shoot length, as well as effective results against bacteria. Additionally, the treatment with ZnO NPs showed excellent MB dye remediation activity. This study underscores the efficacy of endophyte-assisted NPs synthesis with multifunctional properties. However, some limitations of the study, such as TEM analysis, MIC, and MBC for antibacterial studies against plant pathogenic or clinically relevant multidrug-resistant bacteria, can be considered for future work. Further research is required to determine the effectiveness of these synthesized nanoparticles as nanofertilizers in other crop plants, as the results gained here for one crop plant cannot be extended to others. Furthermore, thorough cytotoxicity and ecotoxicity experiments must be performed to verify safety against non-target organisms before any further application is considered. Other future research could focus on their use in pollutant removal procedures. For a better understanding, in vitro and in vivo applications as an anticancer agent can be further explored to study its potential applications in diverse sectors.

## Supporting information

S1 File Raw data. (XLSX)
